# Enhancing the Quality of Hierarchic Relations in the National Cancer Institute Thesaurus to Enable Faceted Query of Cancer Registry Data

**DOI:** 10.1200/CCI.19.00124

**Published:** 2020-05-06

**Authors:** Licong Cui, Rashmie Abeysinghe, Fengbo Zheng, Shiqiang Tao, Ningzhou Zeng, Isaac Hands, Eric B. Durbin, Lori Whiteman, Lyubov Remennik, Nicholas Sioutos, Guo-Qiang Zhang

**Affiliations:** ^1^ School of Biomedical Informatics, University of Texas Health Science Center at Houston, Houston, TX; ^2^ Department of Computer Science, University of Kentucky, Lexington, KY; ^3^ Department of Neurology, McGovern School of Medicine, University of Texas Health Science Center at Houston, Houston, TX; ^4^ Kentucky Cancer Registry, Lexington, KY; ^5^ Division of Biomedical Informatics, Department of Internal Medicine, University of Kentucky, Lexington, KY; ^6^ Enterprise Vocabulary Services, Center for Biomedical Informatics & Information Technology, National Cancer Institute, Bethesda, MD

## Abstract

**PURPOSE:**

To audit and improve the completeness of the hierarchic (or is-a) relations of the National Cancer Institute (NCI) Thesaurus to support its role as a faceted system for querying cancer registry data.

**METHODS:**

We performed quality auditing of the 19.01d version of the NCI Thesaurus. Our hybrid auditing method consisted of three main steps: computing nonlattice subgraphs, constructing lexical features for concepts in each subgraph, and performing subsumption reasoning with each subgraph to automatically suggest potentially missing is-a relations.

**RESULTS:**

A total of 9,512 nonlattice subgraphs were obtained. Our method identified 925 potentially missing is-a relations in 441 nonlattice subgraphs; 72 of 176 reviewed samples were confirmed as valid missing is-a relations and have been incorporated in the newer versions of the NCI Thesaurus.

**CONCLUSION:**

Autosuggested changes resulting from our auditing method can improve the structural organization of the NCI Thesaurus in supporting its new role for faceted query.

## INTRODUCTION

The Kentucky Cancer Registry (KCR)^1^ was established in 1991 at the University of Kentucky Markey Cancer Center (MCC). It is a central cancer registry receiving data about new cancer cases from all health care facilities and physicians in Kentucky within 4 months of diagnosis, as required by state law. Despite advances in cancer research over the last several decades, the cancer burden in Kentucky remains severe. According to State Cancer Profiles statistics provided by the National Cancer Institute (NCI) and the Centers for Disease Control and Prevention, Kentucky is the state that has the nation’s highest cancer burden.^2,3^ In 2000, KCR became a part of the NCI SEER program.^4,5^ The SEER registries are considered to be among the most accurate and complete population-based cancer registries in the world that include stage of cancer at the time of diagnosis and patient survival data.

**CONTEXT**

**Key Objective**
This report aims to improve the structural organization of the National Cancer Institute (NCI) Thesaurus to support its new role as faceted query interface for cancer registries. Distinct from other existing approaches, our method automatically generated suggested changes leveraging both nonlattice subgraphs and lexical features.
**Knowledge Generated**
With our nonlattice subgraph and lexical–based method, potentially missing is-a relations were systematically uncovered. Uncovered missing is-a relations were validated by domain experts and have been incorporated into newer versions of the NCI Thesaurus.
**Relevance**
Enhanced organization of the NCI Thesaurus improves the precision and recall of cohorts of patients with cancer specified using faceted query based on the NCI Thesaurus.

Such cancer registry data have enabled Web-based access to the data and analytic tools for cancer research. For example, State Cancer Profiles provide a user-friendly interface for finding cancer statistics for specific states and counties for public health officials and policymakers. KCR has also developed an NCI-funded Apple iOS app called Cancer Rates^6^ to make incidence and mortality information available on mobile devices. However, the interfaces of such query engines do not support sophisticated data exploration, such as identifying patient cohorts for the feasibility of clinical trials, and have not achieved usability levels approaching those of consumer Web sites, in critical part because of the lack of faceted capabilities.^7-9^ Faceted organization and presentation of metadata are the key mechanisms that allow consumers of Web sites such as Amazon to quickly narrow down from millions of products to items of interest using dimensions of attributes (eg, simple facets such as size, color, maker, price range). Faceted systems for querying clinical data are not widely available because of the complexity of data and the mismatch between the ontologies used for organizing and annotating clinical data (eg, NCI Thesaurus^10,11^) and the desired facet structures and properties. Therefore, in this NCI-funded project, we aimed to overcome these challenges and develop OncoSphere, a faceted query engine using the NCI Thesaurus as a nested facet system (NFS)^12^ to provide Web-based exploration of the KCR data.

A nested facet is a facet that includes a collection of other facets (or subfacets) as its components.^12^ An NFS is a set of nested facets with a hierarchic (or subtype or is-a) relation among them. The efficacy of an NFS requires the properties of soundness and completeness.^12^ Soundness means that all items within each facet are relevant to the facet; that is, for each facet, all the subfacets listed within that facet are indeed its subtypes. Completeness means that any subfacets relevant to a specific facet are already contained in and accessible through the facet; that is, there are no missing subtypes for the facet. The soundness and completeness properties of facets directly affect the performance of the query engine in terms of precision and recall. Incomplete facets will reduce recall, and unsound facets will reduce precision. For instance, “anaplastic T-lymphocyte” is currently not listed as one of the subtypes of “neoplastic large T-lymphocyte” (ie, incomplete facet) in the NCI Thesaurus and would thus be a missing choice in the corresponding facet for “neoplastic large T-lymphocyte.” As a consequence, patients with anaplastic T-lymphocytes would not be included in a cohort of patients with neoplastic large T-lymphocytes, reducing the query recall. Because OncoSphere relies on the hierarchic structure of the NCI Thesaurus for its faceted query interface, it is essential to ensure the quality of the NCI Thesaurus.

In this report, we focus on a quality auditing of the hierarchic structure of the NCI Thesaurus. We developed a hybrid method leveraging a specific substructure called nonlattice subgraph^13^ and lexical features of concepts in the nonlattice subgraph to automatically detect missing hierarchic relations in the NCI Thesaurus. The key idea of our method is that nonlattice subgraphs pinpoint problematic areas that are likely to contain hierarchic quality issues, and lexical features facilitate the identification of potentially missing hierarchic relations in the nonlattice subgraphs through subsumption reasoning.

## METHODS

We used the 19.01d version of the NCI Thesaurus. Our hybrid method consisted of the following steps: computing nonlattice subgraphs, constructing lexical features, and performing subsumption reasoning.

### Computing Nonlattice Subgraphs

**FIG 1.**
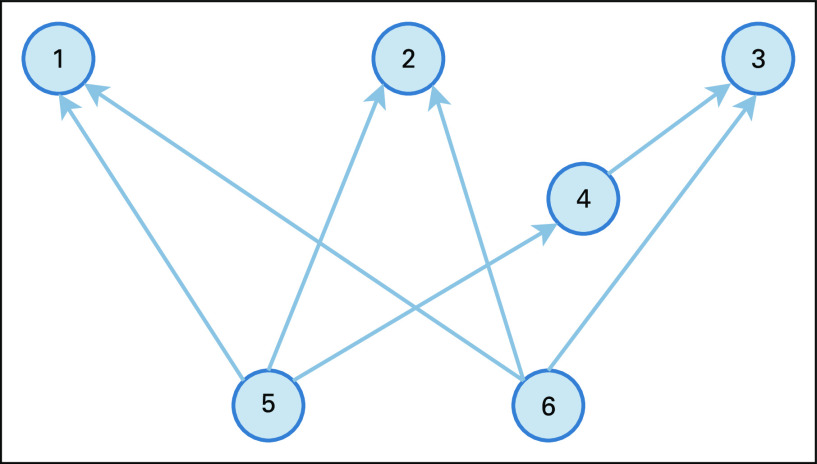
Example of a nonlattice subgraph.

Concepts in the NCI Thesaurus are hierarchically organized as a direct acyclic graph (DAG), where a node (or concept) may have multiple parents. A pair of concepts is called a nonlattice pair, if the two concepts share more than one maximal common descendant (or one minimal common ancestor).^14^ Here the maximal common descendant of two concepts *v* and *w* in a DAG is the highest node that has both *v* and *w* as ancestors, and the minimal common ancestor of two concepts in a DAG is the lowest node that has both concepts as descendants. For instance, in Figure 1, concept 1 and concept 2 have two maximal common descendants, concept 5 and concept 6; therefore, concepts 1 and 2 form a nonlattice pair. Similarly, concept 2 and concept 3 have two maximal common descendants, 5 and 6, and form a nonlattice pair.

A nonlattice pair *P* determines a nonlattice subgraph, which can be obtained by first computing the maximal common descendants of the nonlattice pair, denoted as *mcd*(*P*); reversely computing *mcd*(*P*)’s minimal common ancestors, denoted as *mca*(*mcd*(*P*)); and then aggregating the concepts and relations between (and including) any concept in *mca*(*mcd*(*P*)) and any concept in *mcd*(*P*).^13^ For instance, given the nonlattice pair *P* = (1, 2) in Figure 1, P’s maximal common descendants *mcd*(*P*) are 5 and 6; computing *mcd*(*P*)’s minimal common ancestors obtains 1, 2, and 3; by aggregating concepts between {1, 2, 3} and {5, 6}, we have the nonlattice subgraph containing six concepts {1, 2, 3, 4, 5, 6}.

**FIG 2.**
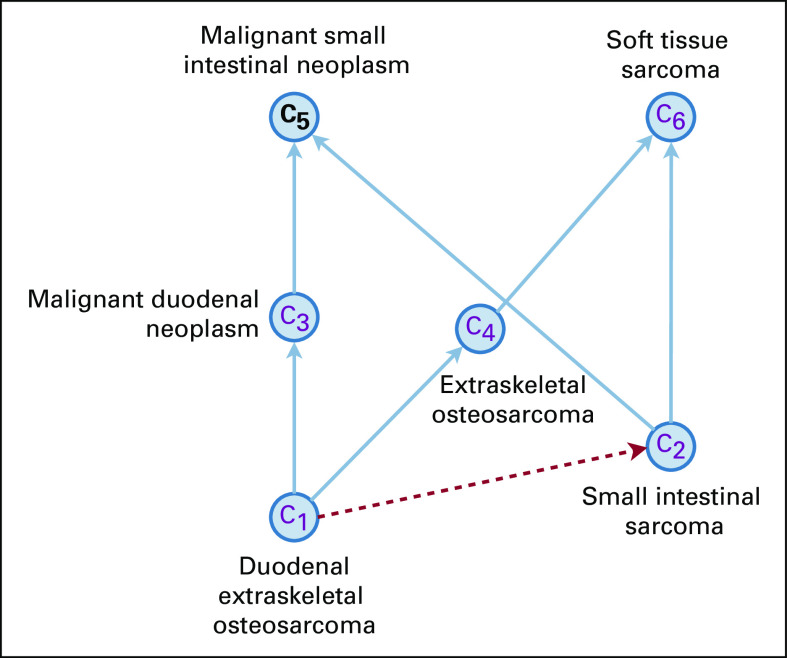
Example of nonlattice subgraph in the NCI Thesaurus (19.01d version).

Figure 2 shows an example of nonlattice subgraph in the NCI Thesaurus determined by the nonlattice pair *P =* (*C*_5_, *C*_6_). *P*’s maximal common descendants *mcd*(*P*) are *C*_1_ and *C*_2_; computing *mcd*(*P*)’s minimal common ancestors still obtains *C*_5_ and *C*_6_, that is, *P* itself; and aggregating concepts in between results in the nonlattice subgraph consisting of six concepts {*C*_1_, *C*_2_, *C*_3_, *C*_4_, *C*_5_, *C*_6_}.

We used an efficient algorithm developed in our previous work^15^ to exhaustively compute all the nonlattice subgraphs in the NCI Thesaurus. This algorithm has been tested on large biomedical terminologies in a DAG, including SNOMED CT, Gene Ontology, and NCI Thesaurus.

### Constructing Lexical Features

We created a set of lexical features (or lexical set) for each concept in the nonlattice subgraph. Given a concept *C* in a nonlattice subgraph *G*, we modeled its lexical set as the words (unordered) appearing in the name of the concept *C* and inherited from the names of *C*’s ancestors in *G*. That is, the concept *C*’s lexical features consist of two parts, where the first part contains the words appearing in the concept *C*’s own name, and the second part contains the words inherited from the names of the concept *C*’s ancestors within the nonlattice subgraph *G*. For example, for concept *C*_1_ = “duodenal extraskeletal osteosarcoma” in Figure 2, the first part of the lexical features contains the words in its own name (ie, {duodenal, extraskeletal, osteosarcoma}). The second part contains the words inherited from *C*_1_’s ancestors (*C*_3_, *C*_4_, *C*_5_, and *C*_6_; ie, {malignant, duodenal, neoplasm, extraskeletal, osteosarcoma, malignant, small, intestinal, neoplasm, soft, tissue, sarcoma}). Because we modeled the lexical features of a concept as a set of words, removing duplicated words in both parts obtains {duodenal, extraskeletal, osteosarcoma, malignant, neoplasm, small, intestinal, soft, tissue, sarcoma}, where “duodenal,” “extraskeletal,” and “osteosarcoma” appear in the name of the concept *C*_1_ itself; “malignant” and “neoplasm” are from its ancestors *C*_3_ and *C*_5_; “small” and “intestinal” are from its ancestor *C*_5_; and “soft,” “tissue,” and “sarcoma” are from its ancestor *C*_6_. Table 1 lists the lexical sets of all concepts in the nonlattice subgraph, shown in Figure 2.

**TABLE 1.**
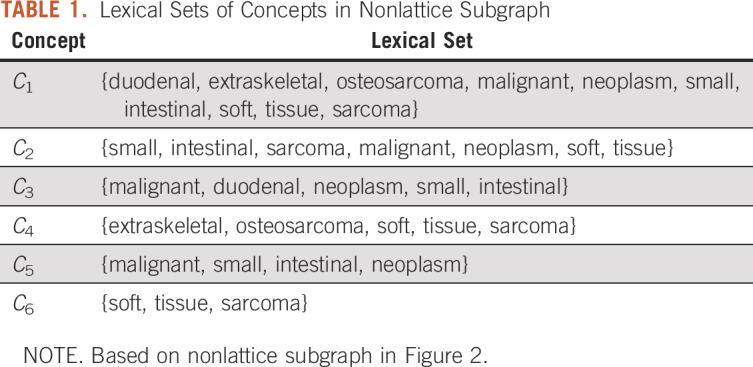
Lexical Sets of Concepts in Nonlattice Subgraph

### Performing Subsumption Reasoning

We performed subsumption reasoning to detect potentially missing is-a relations among the pairs of concepts currently not hierarchically related. For each nonlattice subgraph, we first identified pairs of concepts currently not hierarchically related; then, for each pair of concepts (eg, *C*_1_ and *C*_2_), we checked whether their lexical sets had an inclusion relation as follows: if *C*_2_’s lexical set is a proper subset of *C*_1_’s lexical set, we suggest a potentially missing is-a relation between *C*_1_ and *C*_2_ (ie, *C*_1_ is-a *C*_2_); if *C*_1_’s lexical set is a proper subset of *C*_2_’s lexical set, we suggest a potentially missing is-a relation between *C*_2_ and *C*_1_ (ie, *C*_2_ is-a *C*_1_); otherwise, no suggestion will be made.

For instance, for concepts *C*_1_ (duodenal extraskeletal osteosarcoma) and *C*_2_ (small intestinal sarcoma) in Figure 2, because *C*_2_’s lexical set {small, intestinal, sarcoma, malignant, neoplasm, soft, tissue} is a proper subset of *C*_1_’s lexical set {duodenal, extraskeletal, osteosarcoma, malignant, neoplasm, small, intestinal, soft, tissue, sarcoma}, we suggest *C*_1_ is-a C_2_; that is, “duodenal extraskeletal osteosarcoma” is a subtype of “small intestinal sarcoma” (dashed red arrow in Fig 2). For concepts *C*_5_ (malignant small intestinal neoplasm) and *C*_6_ (soft tissue sarcoma) in Figure 2, there is no inclusion between their lexical sets, and therefore, no suggestion will be made for this pair of concepts.

When performing such subsumption reasoning, we did not make suggestions for certain scenarios prone to generate incorrect suggestions, such as concepts containing stop words (eg, “and/or,” “no,” “not,” “without,” “except,” “by”) and lexical sets containing antonyms (eg, “small,” “large”). In addition, after obtaining all the potential missing is-a relations in nonlattice subgraphs, we further removed redundant is-a relations that could be inferred by other is-a relations.

## RESULTS

**FIG 3.**
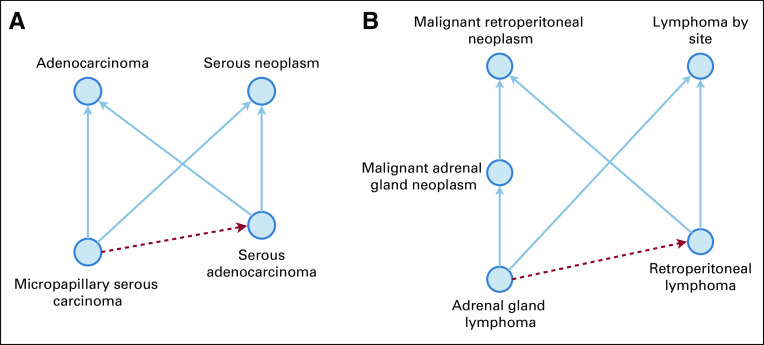
(A) Nonlattice subgraph of size four suggesting a missing is-a relation: “micropapillary serous carcinoma” is-a “serous adenocarcinoma.” (B) Nonlattice subgraph of size five suggesting a missing is-a relation: “adrenal gland lymphoma” is-a “retroperitoneal lymphoma.”

Using the 19.01d version of the NCI Thesaurus, a total of 9,512 nonlattice subgraphs were obtained. The sizes of the nonlattice subgraphs ranged from four to 644. Our hybrid method detected 925 potentially missing is-a relations in 441 nonlattice subgraphs. Figure 3 shows two examples of the identified missing is-a relations in two nonlattice subgraphs sized four and five, respectively.

For evaluation, we provided the NCI Enterprise Vocabulary Services (EVS), which manages the NCI Thesaurus, with 253 potentially missing is-a relations in nonlattice subgraphs of ≤ 15 in size. These nonlattice subgraphs were visualized in PDFs and organized in terms of the subhierarchies to facilitate the EVS experts’ review and evaluation. Table 2 lists the number of potentially missing is-a relations identified by our method according to the subhierarchies. The EVS experts reviewed four subhierarchies: “disease, disorder or finding,” “activity,” “abnormal cell,” and “anatomic structure, system, or substance.” The subhierarchy “disease, disorder or finding” contained 136 potentially missing is-a relations, among which 50 were verified as valid by EVS experts. In total, 72 of 176 reviewed samples were confirmed as valid missing is-a relations and have been incorporated into the newer versions of the NCI Thesaurus. Table 3 lists 10 examples of valid missing is-a relations verified by EVS experts (Data Supplement provides a comprehensive list).

**TABLE 2.**
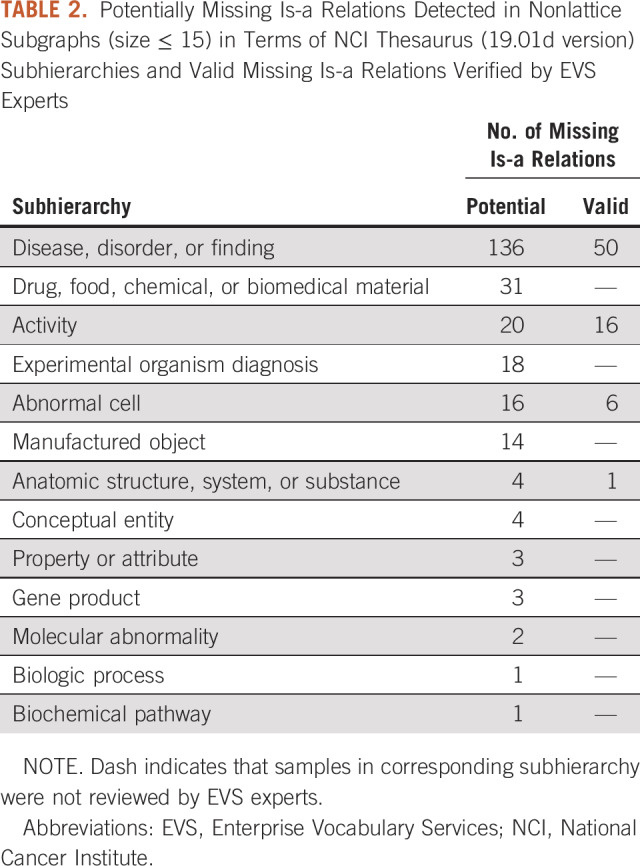
Potentially Missing Is-a Relations Detected in Nonlattice Subgraphs (size ≤ 15) in Terms of NCI Thesaurus (19.01d version) Subhierarchies and Valid Missing Is-a Relations Verified by EVS Experts

**TABLE 3.**
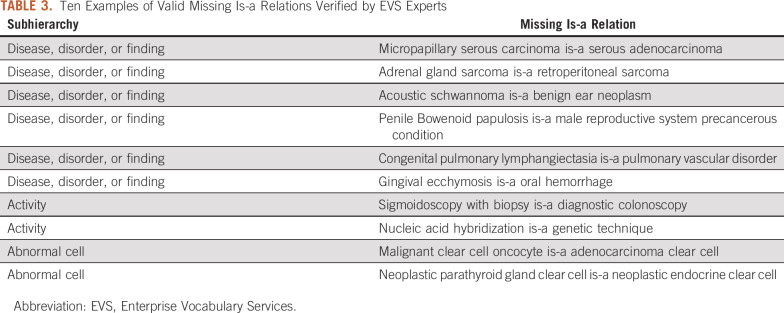
Ten Examples of Valid Missing Is-a Relations Verified by EVS Experts

## DISCUSSION

Although our hybrid method was able to suggest valid missing is-a relations, it sometimes made erroneous suggestions for ambiguous cases. For instance, our method suggested “metastatic malignant neoplasm in the pancreas” was a subtype of “metastatic malignant pancreatic neoplasm.” This suggestion was invalid, because the latter concept refers to metastatic malignant neoplasms that originate from the pancreas and spread to other anatomic sites. This was an erroneous pattern of “malignant neoplasm in x site” versus “malignant x site neoplasm” that our lexical set–based method was not able to differentiate. A potential solution to avoid such erroneous suggestions would be to add “in” as a stop word. However, adding it as a stop word would miss valid suggestions, such as “metastatic malignant neoplasm in the sellar region” is-a “metastatic malignant neoplasm in the central nervous system.” This illustrates the challenge of the varying degrees to which different stop words generate erroneous suggestions. For future improvement, we plan to explore machine learning–based approaches to train the model on positive and negative samples for different stop words and test whether the model can differentiate the degrees or even automatically learn new stop words in addition to what we have used. Another potential solution would be to leverage the roles defining the concepts, thus automatically facilitating the subsumption reasoning and differentiating the ambiguous concepts.

**FIG 4.**
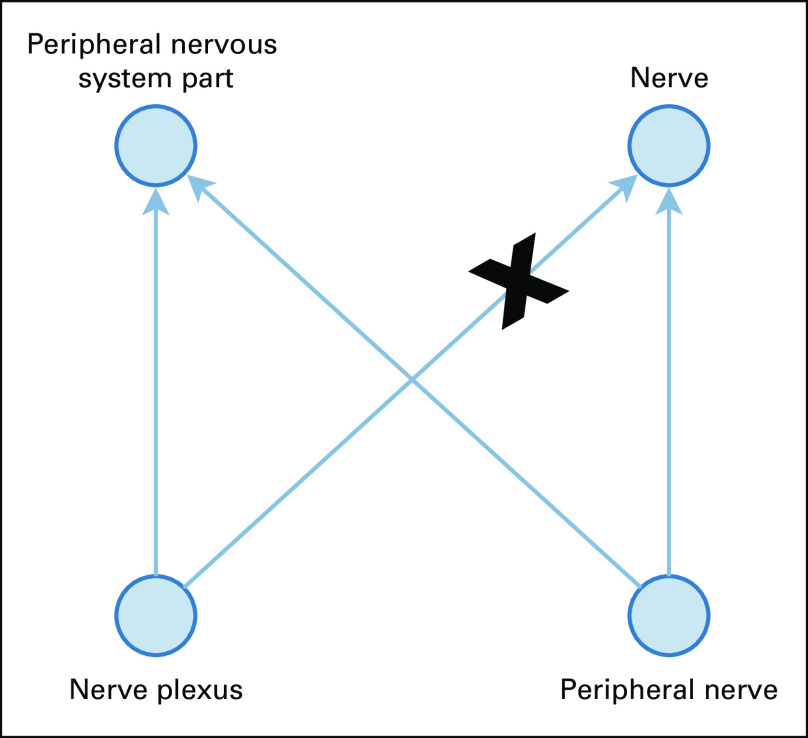
Example of scenarios where erroneous suggestions further reveal problematic existing relations.

For certain scenarios, erroneous suggestions made by our method further reveal problematic relations existing in the NCI Thesaurus. For example, our method suggested the following invalid relation: “nerve plexus” is-a “peripheral nerve.” However, the existing relations leveraged by our method to generate the suggestion were: “nerve plexus” is-a “peripheral nervous system part” and “nerve plexus” is-a “nerve” (as shown in the nonlattice subgraph in Fig 4). Because nerve plexus has peripheral nerves as parts but is not itself a nerve, this invalid suggestion revealed an incorrect existing relation in the NCI Thesaurus: “nerve plexus” is-a “nerve” the link with an X in Fig 4). This indicates that our method may help with further identification of problematic is-a relations in the NCI Thesaurus in addition to missing is-a relations.

In our previous work,^16^ we used six predefined lexical patterns in nonlattice subgraphs to identify potentially missing is-a relations in the NCI Thesaurus. In this work, we directly used the lexical sets of concepts to perform subsumption reasoning, with no need to predefine lexical patterns. More importantly, our method in this work identified previously undiscovered missing is-a relations in the NCI Thesaurus. In another related work,^17^ we leveraged nonlattice subgraphs and concept names to perform subsumption reasoning for suggesting potentially missing is-a relations in SNOMED CT. In this work, we modeled the lexical features of a concept in the NCI Thesaurus using not only the name of the concept itself but also the names of the concept’s ancestors.

Although our hybrid method was capable of revealing valid missing is-a relations, it only touched upon a small portion of nonlattice subgraphs in the NCI Thesaurus (441 of 9,512), leaving the remaining nonlattice subgraphs untapped. New methods are needed to uncover the potential quality issues in the untapped nonlattice subgraphs. We will further leverage the roles defining the concepts to facilitate the quality auditing task. Regarding the use of stop words in our method, although it can avoid making erroneous suggestions, it may also miss valid suggestions. Additional research is needed to specifically handle concepts containing stop words.

Another limitation is that our evaluation was preliminary in two aspects. First, the evaluation was based on nonlattice subgraphs ≤ 15 in size, because the EVS experts would be visually overwhelmed with large graphs to manually review. Second, the EVS experts did not review all the provided potentially missing is-a relations. We plan to provide EVS experts with a random sample of potentially missing is-a relations detected from a newer version of the NCI Thesaurus after improving the ability of our method to distinguish ambiguous concepts.

In conclusion, we developed a hybrid method to automatically suggest potentially missing is-a relations in the NCI Thesaurus. Autosuggested changes resulting from our auditing method can improve the structural organization of the NCI Thesaurus in supporting its new role for faceted query.
